# Molecular characterization of the symbionts associated with marine nematodes of the genus *Robbea*‡

**DOI:** 10.1111/j.1758-2229.2009.00019.x

**Published:** 2009-04

**Authors:** Christoph Bayer, Niels R Heindl, Christian Rinke, Sebastian Lücker, Joerg A Ott, Silvia Bulgheresi

**Affiliations:** 1 Departments of Marine Biology, University of ViennaAlthanstrasse 14, 1090 Vienna, Austria; 2 Departments of Microbial Ecology, University of ViennaAlthanstrasse 14, 1090 Vienna, Austria

## Abstract

Marine nematodes that carry sulfur-oxidizing bacteria on their cuticle (*Stilbonematinae, Desmodoridae*) migrate between oxidized and reduced sand layers thereby supplying their symbionts with oxygen and sulfide. These symbionts, in turn, constitute the worms' major food source. Due to the accessibility, abundance and relative simplicity of this association, stilbonematids may be useful to understand symbiosis establishment. Nevertheless, only the symbiont of *Laxus oneistus* has been found to constitute one single phylotype within the *Gammaproteobacteria*. Here, we characterized the symbionts of three yet undescribed nematodes that were morphologically identified as members of the genus *Robbea*. They were collected at the island of Corsica, the Cayman Islands and the Belize Barrier Reef. The surface of these worms is covered by a single layer of morphologically undistinguishable bacteria. 18S rDNA-based phylogenetic analysis showed that all three species belong to the *Stilbonematinae*, although they do not form a distinct cluster within that subfamily. 16S rDNA-based analysis of the symbionts placed them interspersed in the cluster comprising the sulfur-oxidizing symbionts of *L. oneistus* and of marine gutless oligochaetes. Finally, the presence and phylogeny of the *aprA* gene indicated that the symbionts of all three nematodes can use reduced sulfur compounds as an energy source.

## Introduction

Marine nematodes that live a few centimetres below the surface of sandy bottoms may carry sulfur-oxidizing bacteria (SOB) within their body as endosymbionts [*Astomonema* (Ott *et al.*, 1982; Vidakovic and Boucher 1987; Giere *et al.*, 1995; Musat *et al.*, 2007) and *Parastomonema* (Kito, 1989)] or on their surface as ectosymbionts. The latter belong to the subfamily *Stilbonematinae* and consist of the genera *Adelphus* Ott 1997, *Catanema* Cobb 1920, *Eubostrichus* Greef 1869, *Laxus* Cobb 1894, *Leptonemella* Cobb 1920, *Robbea*Gerlach 1956, *Squanema*Gerlach 1963 and *Stilbonema* Cobb 1920 (reviewed in Ott *et al.*, 2004a,b). The worms migrate between oxygenated, upper sand layers and anoxic, sulfidic, deeper ones (Ott *et al.*, 1991) allowing the bacteria to obtain the oxygen they need as e^-^ acceptor and the sulfur compounds (e.g. hydrogen sulfide, thiosulfate) as e^-^ donor (Polz *et al.*, 1992; Hentschel *et al.*, 1999). Stable carbon isotope incorporation experiments showed that the ectosymbionts are the major components of their host diet (Ott *et al.*, 1991).

Symbionts are probably acquired from the environment because unhatched early embryos of *Laxus oneistus* are symbiont-free (Silvia Bulgheresi and Joerg A. Ott, in preparation). Environmental transmission would also enable nematodes to re-establish their symbiotic coat every time they replace their cuticle with a newly synthesized one. This process, known as molting or ecdysis, occurs several times during worm development. Moreover, *Robbea* sp.1 and sp.3 symbiont 16S rDNAs were detected in sand and seawater by polymerase chain reaction (PCR) and fluorescence *in situ* hybridization (FISH) with 16S rRNA-specific primers. As for the mechanisms of symbiont recruitment from the environment, we showed that the Ca^2+^-dependent lectin Mermaid mediates symbiont–symbiont and worm–symbiont attachment in *L. oneistus* (Bulgheresi *et al.*, 2006).

Up to the present study only the symbionts of *L. oneistus* have been shown to belong to one single phylotype of *Gammaproteobacteria* closely related to the endosymbionts of marine gutless oligochaetes (Polz *et al.*, 1994) and of *Astomonema* sp. (Musat *et al.*, 2007). Although a molecular characterization of the large, multinucleated, filamentous symbiont of *Eubostrichus dianae* has been attempted, its 16S rDNA could not be amplified by PCR (Polz *et al.*, 1999).

In this study, we molecularly characterized three associations involving stilbonematids which we assigned to the genus *Robbea* (Gerlach, 1956; 1963) based on their morphological characteristics. We collected *Robbea* sp.1 in the Mediterranean Sea from a subtidal sand patch close to a *Posidonia oceanica* seagrass meadow near Calvi (Corsica, France), and *Robbea* sp.2 and *Robbea* sp.3 in the Caribbean Sea from shallow back-reef sandbars at Little Cayman Island (Cayman Islands) and Carrie Bow Cay (Belize) respectively. We first analysed the phylogenetic position of the worms by making clone libraries of their 18S rRNA genes. We then characterized the symbionts associated with each species by cloning their respective 16S rRNA genes. To confirm that the latter were indeed derived from the ectosymbionts, we applied FISH on whole worms. Finally, the cloning and phylogenetic analysis of a gene that is involved in sulfur metabolism support the sulfur-oxidizing nature of the *Robbea* symbionts.

## Results and discussion

### Morphological and 18S rDNA-based molecular characterization of *Robbea* nematodes

**Fig. 1:**
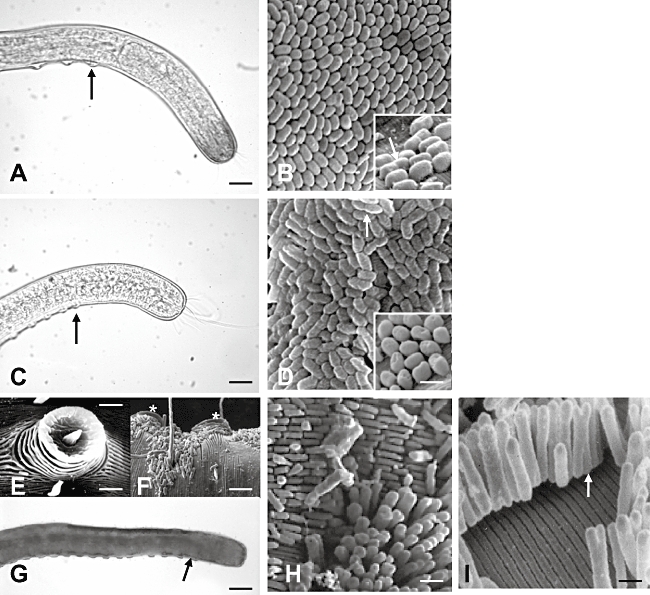
Photomicrographs of the anterior regions of fixed *Robbea* sp.1 (A), *Robbea* sp.2 (C) and *Robbea* sp.3 (G) and scanning electron microscopy (SEM) photographs of their respective symbionts (B, D, H and I). Black arrows point to the beginning of the suckers' row on each worm in (A), (C) and (G), while white arrows point to dividing symbionts in (B), (D) and (I). (E) and (F) are SEM photographs of one individual bacteria-free sucker, and two symbiont-coated suckers (asterisks) of *Robbea* sp.3, respectively. Scale bar is: 25 µm in (A) and (C); 1.5 µm in (B) and (D); 3 µm in (E); 8 µm in (F); 40 µm in (G); 2 µm in (H); 0.6 µm in (I).

**Fig. 2:**
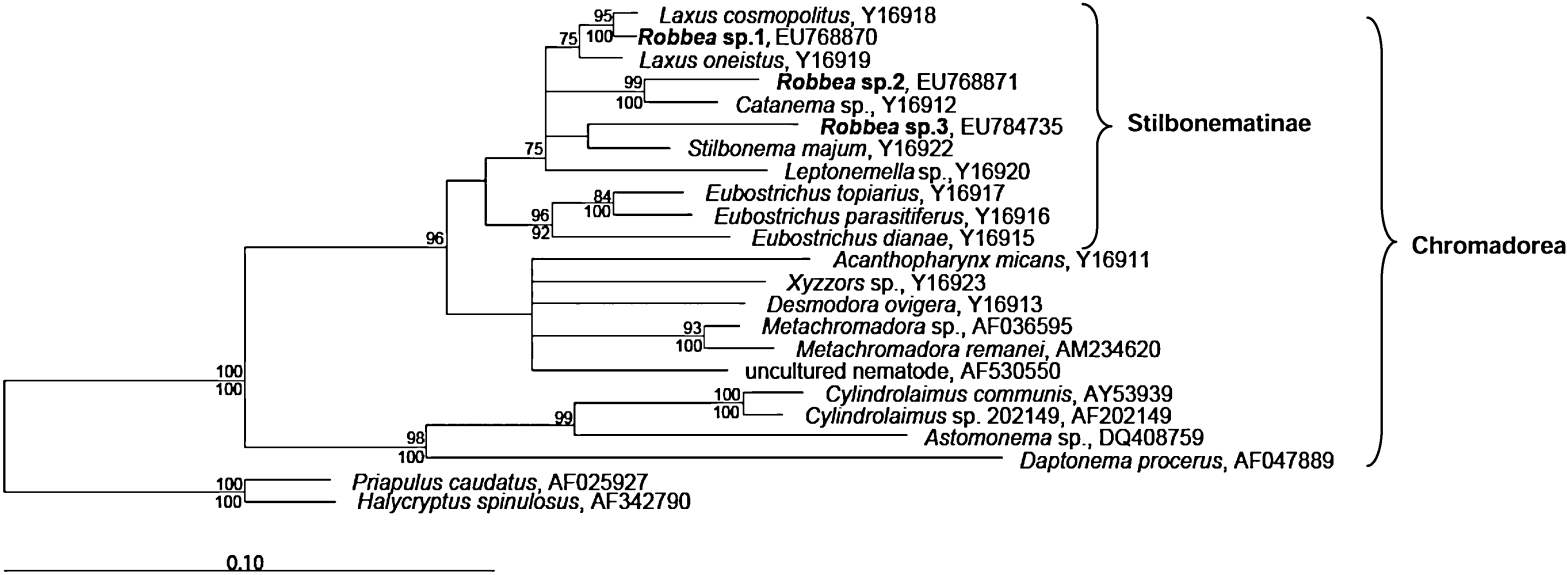
18S rDNA-based consensus phylogenetic tree based on maximum parsimony and Treepuzzle analysis showing the relationship of the *Robbea* worms (in bold) with other *Stilbonematinae* and other *Chromadorea*. Treepuzzle support values are depicted above the respective branches and maximum parsimony bootstrap values below the branches. Only Treepuzzle support values above 75% and parsimony bootstrap values higher than 70% are displayed. The scale bar represents 10% estimated sequence divergence.

The genus *Robbea* was established by Gerlach (1956). It is characterized by a clearly set off and muscle-rich distal part (corpus) of the tripartite pharynx. Moreover, all males, except in *Robbea caelestis*, are provided by a row of ventromedian suckers in the postpharyngeal region which are supposed to be copulation-helping organs (J.A. Ott, unpubl. data; Fig. 1A, C, E and G and asterisks in Fig. 1F). The number of suckers is constant and species-specific. Because each of the three nematodes characterized in this study had a tripartite, muscle-rich pharynx and carried a row of ventromedian suckers, we assigned them to the genus *Robbea*. Nevertheless, they did not form a monophyletic lineage within the *Stilbonematinae* (*Chromadorea*) in our 18S rDNA-based phylogenetic reconstruction (Fig. 2). It is therefore conceivable that their distinctive morphological traits evolved several times independently. Alternatively, supplementary sequence information from the 28S or Internal Transcribed Spacers (ITS) rDNA or from mitochondrial genes might be needed to support the genus *Robbea* at the molecular level.

*Robbea* sp.3 and *Stilbonema majum* were the only stilbonematids which showed the highest 18S rDNA sequence similarity with one another, while co-occurring in the same collection site, the Belize Barrier Reef. The 18S rDNAs of *Robbea* sp. 2, however, showed the highest sequence similarity with that of stilbonematids collected in an extremely distant geographical location.

Each nematode is covered by a single morphotype of symbionts: *Robbea* sp.1 and sp.2 display coccoid bacteria *c*. 1.5 µm wide (Fig. 1B and D respectively) whose shape and arrangement are reminiscent of kernels on a corn cob. *Robbea* sp.3 is covered by spindle-shaped rods *c*. 2 µm long (Fig. 1F). These assume different orientations with respect to the worm's surface, with some standing perpendicularly, as observed for *L. oneistus*, and some laying horizontally. In *Robbea* sp.1 and sp.2, the symbionts appear to divide transversally (arrows in Fig. 1B and D respectively). In *Robbea* sp.3 they divide longitudinally (arrows in Fig. 1H and I), a special mode of binary fission also exhibited by *L. oneistus* symbionts (Polz *et al.*, 1992; 1994). Concerning the length of the microbial coat, only the anterior-most region of *Robbea* sp.3 and the very tip of the tail are symbiont-free. In *Robbea* sp.1 and *Robbea* sp.2, instead, the coat starts a short distance behind the anterior end, coinciding with a reduction in the worm diameter to accommodate the symbionts. This last feature is also displayed by *L. oneistus*. As in all other known stilbonematids, the *Robbea* symbionts are densely packed and appear bright white in incident light, probably due to inclusions of elemental sulfur (Himmel *et al.*, 2009).

### *Robbea* symbionts belong to the marine nematode and oligochaete symbionts cluster

**Fig. 3:**
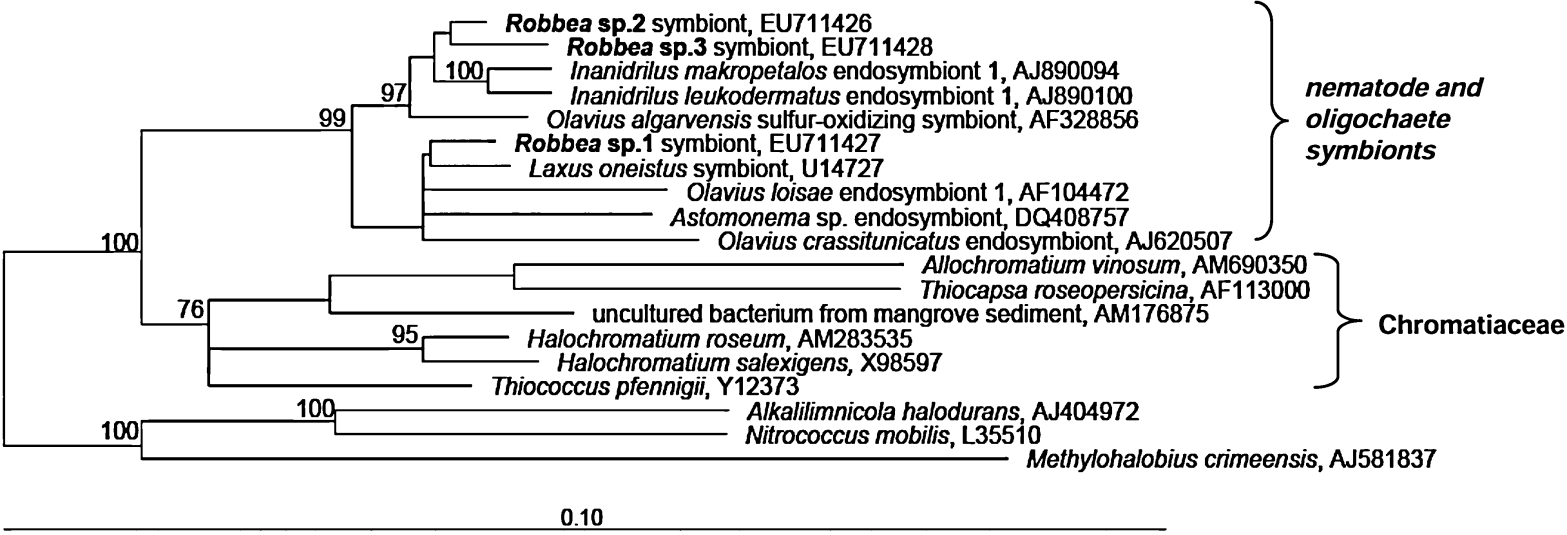
16S rDNA-based phylogenetic tree based on Treepuzzle analysis showing the relationship of the *Robbea* symbionts (in bold) with other stilbonematid and oligochaete symbionts, as well as other bacteria belonging to the *Chromatiaceae* and other vestimentiferan and mussel symbionts. Treepuzzle support values are depicted above the respective branches and maximum parsimony bootstrap values below the branches. Only Treepuzzle support values above 75% are displayed. The scale bar represents 10% estimated sequence divergence.

*Robbea* symbiont 16S rDNA clones were randomly picked and comparison of their complete sequences showed that they could be assigned to three distinct clone groups belonging to the *Gammaproteobacteria*, with a sequence similarity within each clone group ≥ 99.8%. In our 16S rDNA-based phylogenetic reconstruction (Fig. 3) the three obtained gammaproteobacterial 16S rDNAs clustered with those of the symbionts of *L. oneistus*, of the nematode *Astomonema* sp., and of all known marine gutless oligochaetes (*Inanidrilus* and *Olavius* spp.). This nematode–oligochaete symbiont cluster is most closely related to the SOB from the family *Chromatiaceae* (> 90%). It is intriguing that, although free-living, some of these sulfur purple bacteria engage in symbiotic associations with unrelated bacteria in phototrophic consortia (Tonolla *et al.*, 2000; Overmann, 2002).

Our phylogenetic reconstruction shows that the three *Robbea* symbionts (16S rDNA sequence identity ≥ 97.1%) do not form a distinct group within the nematode–oligochaete sulfur-oxidizing symbionts cluster (16S rDNA sequence identity ≥ 95.4%). Moreover, nematode symbionts cannot be consistently grouped according to the geographical origin of their hosts and probably did not speciate in concert with their hosts. Phylogenetic incongruence between host and symbiont is typical of horizontally transmitted symbioses (Moran and Baumann, 2000), and was also observed for marine gutless oligochaetes and their sulfur-oxidizing symbionts (Dubilier *et al.*, 2001; Blazejak *et al.*, 2006; Musat *et al.*, 2007).

**Fig. 4:**
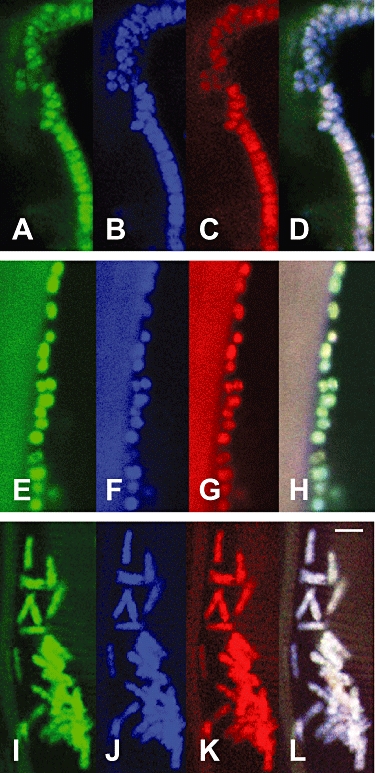
Fluorescence *in situ* hybridization (FISH) confocal microscope photographs of *Robbea* sp.1 (A–D), *Robbea* sp.2 (E–H) and *Robbea* sp.3 (I–L) symbionts attached to the worm surface. Each single symbiont is triple stained with a eubacteria-specific probe (green), a *Gammaproteobacteria*-specific probe (blue), and a symbiont-specific probe (red). (D), (H) and (L) are overlay pictures of (A)–(C), (E)–(G) and (I)–(K), respectively. Scale bar is 2 µm.

To confirm that the gammaproteobacterial 16S rDNA sequences derived from the *Robbea* symbionts, we carried out FISH with the symbiont-specific probes Rca470, Rss457 and Rhs465, for *Robbea* sp.1, sp.2 and sp.3 respectively (Table 1). All the bacteria attached to the worms were triple stained by the eubacterial probe EUB338, by the *Gammaproteobacteria*-specific probe GAM42a and by the respective specific probe (Fig. 4). In contrast, no FISH signal was detectable with the negative control probe NON338 or with a *Betaproteobacteria*-specific probe (data not shown). This indicates that the bacteria covering each of the three *Robbea* species belong to one single phylotype and that no additional bacteria are present. This is consistent with the electron microscopy analysis, which shows only one bacterial morphotype on each *Robbea* worm, and with our highly homogeneous 16S rDNA libraries.

**Table 1:** Probes used for FISH.

Probe	Standard probe name^a^	Specificity	Sequence/5′ modification	Target RNA	Position^b^,^c^	Formamide percentage/ incubation time (h)/probe concentration (ng µl^−1^)	Reference
EUB338	S-*-BactV-0338-a-A-18	Most bacteria	5′-GCT GCC TCC CGT AGG AGT-3′/fluorescein	16S	338–355	35–40%/1.5–o.n./3	Amann *et al.* (1990)
GAM42a	L-C-gProt-1027-a-A-17	*Gammaproteobacteria*	5′-GCC TTC CCA CAT CGT TT-3′/Cy5	23S	1027–1043	35–40%/1.5–o.n./3	Manz *et al.* (1992)
Rcas470	S-Rob1s-0471-a-A-21	*Robbea* sp.1 symbiont	5′-TGC GTA ACG TCA AGA CCC TGG-3′/Cy3	16S	471–491	25%/1.5/3.8	This study
Rss456	S-*-Rob2s-0457-a-A-21	*Robbea* sp.2 symbiont, *Inanidrilus leukodermatus* endosymbiont 1 (GenBank Accession No. AJ890100)	5′-ACC CTG AGC TAT TAA CCC AAG-3′/Cy3	16S	457–477	35%/o.n./4	This study
Rhs465	S-Rob3s-a-A-21	*Robbea* sp.3 symbiont	5′-AAC GTC AGG ATC CCG AGC TAT-3′/Cy3	16S	466–486	40%/3/2.3	This study
NON338	Not named	Negative control	5′-ACT CCT ACG GGA GGC AGC-3′/Cy3	16S	338–355	35–40%/1.5–o.n./3	Wallner *et al.* (1993)
BET42a	L-C-bProt-1027-a-A-17	*Betaproteobacteria*	5′-GCC TTC CCA CTT CGT TT-3′/fluorescein	16S	1027–1043	35–40%/1.5–o.n./3	Manz *et al.* (1992)

a According to Alm and colleagues (1996).

b 16S rRNA position, *Escherichia coli* numbering (Brosius *et al.*, 1978).

c 23S rRNA position, *E. coli* numbering (Brosius *et al.*, 1981).

o.n., overnight.

### *aprA* gene analysis of stilbonematid-associated bacteria

**Fig. 5:**
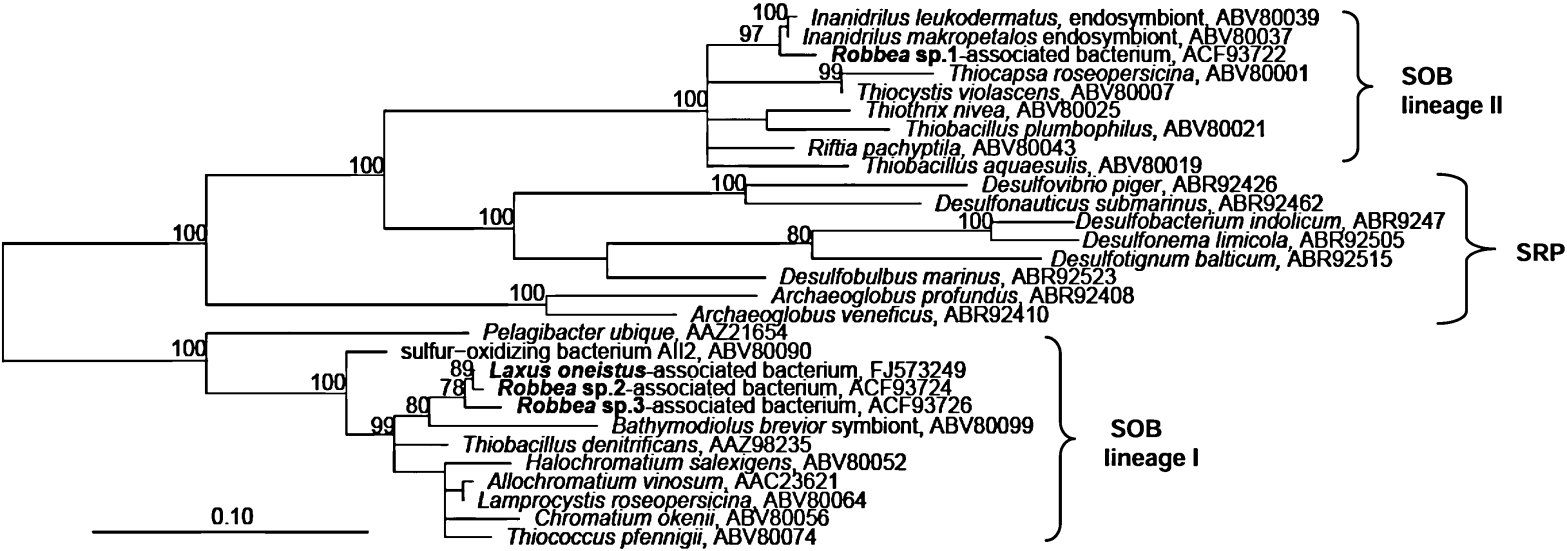
Phylogenetic reconstruction based on Treepuzzle analysis of AprA sequences from the *Robbea*-associated bacteria (in bold). Only support values above 75% are displayed. The scale bar represents 10% estimated sequence divergence.

To gain evidence that *Robbea* symbionts are indeed SOB, we cloned a fragment of the gene encoding for the alpha subunit of the adenosine-5′-phosphosulfate (APS) reductase (*aprA*), an enzyme involved in sulfur metabolism. The AprA protein reduces APS to sulfite in sulfate-reducing bacteria (SRB), but also catalyses the reverse reaction in SOB (Hipp *et al.*, 1997; Sanchez *et al.*, 2001; Friedrich, 2002). By using a set of *aprA*-specific primers, we PCR amplified and cloned a ∼1400-nt-long fragment from *Robbea*- and *L. oneistus*-associated bacteria. Several clones from each *aprA* library were randomly picked (see *Experimental procedures*) and their predicted protein sequences used for tree calculation (Fig. 5).

The AprA sequences of bacteria associated with *Robbea* sp.2, sp.3 and *L. oneistus* clustered together with those of the *Bathymodiolus brevior* symbiont, and of some free-living sulfur-oxidizing gammaproteobacteria and sulfur purple bacteria [AprA-lineage I; see Meyer and Kuever (2007b) for a definition of AprA-lineages I and II]; *Robbea* sp.1-associated symbiont AprA, instead, clustered with those of gutless oligochaete sulfur-oxidizing symbionts (AprA-lineage II). Notably, *Robbea* sp.2 and sp.3 symbionts cluster together in both the 16SrDNA- and *aprA*-based trees.

In conclusion, all the AprA sequences obtained in this study are most closely related to SOB AprAs. This suggests that *Robbea* symbionts oxidize sulfur compounds as an energy source.

## Conclusions

We characterized three new nematode–bacteria associations with very different geographical origins – the island of Corsica, the Cayman Islands and the Belize Barrier Reef. Although we cannot exclude that the three *Robbea* symbionts could stably associate with other marine organisms, our data show that each *Robbea* sp. is always coated by one characteristic symbiont phylotype. The basis of this conclusion is that each 16S rDNA and each *aprA* library was highly homogeneous and that the symbionts of each species were reproducibly stained by a symbiont 16S rDNA-specific FISH probe. Accordingly, electron microscopic analysis revealed that individuals of each *Robbea* sp. are always coated by the same, characteristic bacterial morphotype.

Our 18S rDNA-based tree shows that all three nematode species are stilbonematids, albeit additional worm nuclear and/or mitochondrial DNA sequence information is needed to confirm the genus *Robbea* at the molecular level.

Intriguingly, the 16S rDNAs of the stilbonematid symbionts are tightly grouped with those of mouthless oligochaetes. One explanation is that nematodes and oligochaetes co-occur in shallow-water sandy bottoms and they are all exposed to a similar pool of environmental bacteria. This habitat potentially promoted the establishment of these associations several times in the course of the evolution and at many different geographical locations. In this scenario, nematodes and oligochaetes recruited similar bacteria from this shared habitat as prospective symbionts. Sequencing of one or more stilbonematid symbiont metagenome(s) might unveil molecular adaptations shared by the oligochaete and nematode sulfur-oxidizing symbionts.

The fact that *Robbea*-associated bacteria harbour SOB-like *aprA* genes indicates that they gain energy from oxidation of reduced sulfur compounds. Moreover, their white appearance supports their capacity to store elemental sulfur. Migration of *Robbea* nematodes between deep and superficial sand layers, as observed for *L. oneistus* (Ott *et al.*, 1991), would alternatively supply their symbionts with reduced sulfur compounds and oxygen. In the absence of oxygen, symbionts might use nitrate to respire sulfide (Hentschel *et al.*, 1999), while they could resort to their sulfur stores when sulfide is unavailable in the environment.

In turn, the *Robbea* worms might feed on their symbionts. Stable isotope incorporation experiments and electron microscope analysis of the gut microbiome indicate this to be the case for other stilbonematids (Ott *et al.*, 1991). Cloning of other symbiont genes involved in sulfur metabolism and carbon fixation, and transmission electron microscopy of the symbionts coupled with multi-isotope imaging mass spectrometry will shed light on their physiology.

The geographical distribution of the three *Robbea* nematodes characterized in this study appears to be restricted to the respective collection sites. One future task will be to investigate if the stilbonematid symbionts can be found only in the host habitat, as in the case of tube worms (Harmer *et al.* 2008) and lucinid mussels symbionts (Gros *et al.*, 2003) or, instead, are widely distributed throughout the oceans and can survive without their hosts.

Another key question is how specific ectosymbionts are recruited from the environment by different stilbonematid species. In this respect, we plan to identify which repertoires of Mermaid isoforms are expressed by the *Robbea* worms and to compare them with each other and with those of *L. oneistus*. An exciting outcome could be that expression of a characteristic lectin repertoire by each stilbonematid species underpins acquisition and maintenance of a specific bacterial coat.

## Experimental procedures

### Specimen collection

*Robbea* sp.1 was collected in July 2007 from a subtidal sand patch close to a *P. oceanica* seagrass meadow in *c*. 2 m depth in the harbour of the Station de Recherches Sous-Marines et Océanographiques (STARESO), Calvi, France (42°34′49″N, 8°43′27″W). *Robbea* sp.2 was collected in October 2006 in *c*. 1 m depth from a shallow water back-reef sand bar off Point of Sand Beach on Little Cayman, Cayman Islands (19°42′08″N, 79°57′46″W). *Robbea* sp.3 was collected in November 2007 in *c*. 1 m depth from a shallow water back-reef sand bar off Carrie Bow Cay, Belize (16°48′11″N, 88°04′55″W). The worms were extracted from the sand by shaking it in seawater and pouring the supernatant through a 63-µm-pore-size mesh screen. Single individuals were then picked by hand under a dissecting microscope. *Robbea* sp.1 and *Robbea* sp.3 worms were fixed either in ethanol, for DNA extraction, or in 1% osmium tetroxide in seawater, for FISH (Rinke *et al.*, 2006), and then stored in ethanol at −80°C. *Robbea* sp.2 worms were flash frozen in liquid N_2_ and stored at −80°C either unfixed (for DNA extraction) or upon methanol fixation (for FISH).

### Scanning electron microscopy

Worms were pre-fixed in a 2.5% glutaraldehyde, 0.1 M sodium cacodylate, 2% sucrose solution, rinsed with 0.1 M sodium cacodylate buffer, and post-fixed in a 1% osmium tetroxide, 0.1 M sodium cacodylate, 2% sucrose solution. After alcohol dehydration, worms were gold sputter coated and viewed through a Philips XL 20 scanning electron microscope.

### DNA extraction and PCR amplification of 18S rDNA

We extracted and purified the DNA from single *Robbea* worms as described previously (Schizas *et al.*, 1997) and 2 µl was used as a template for each PCR. A fragment of the *Robbea* sp.1 18S rRNA gene was amplified by PCR with the general eukaryotic primers 1f (5′-CTGGTTGATYCTGCCAGT-3′; Winnepenninckx *et al.*, 1995) and 2023r (5′-GGTTCACCTACGGAAACC-3′; Pradillon *et al.*, 2007). Cycling conditions were 94°C for 4 min; 94°C for 45 s, 49°C for 30 s, 72°C for 1 min 45 s 35×; 72°C for 10 min. The PCR product was 1779 nt. *Robbea* sp.2 18S rRNA was amplified with the general eukaryotic primers 1f (see above) and 18SE (5′-ATGATCCTTCCGCAGGTTCAC-3′; Perotto *et al.*, 2000) and *Robbea* sp.3 18S rRNA was amplified with primers 1f and 2023r. Cycling conditions were: 95°C for 5 min; 95°C for 45 s, 48°C for 45 s, 72°C for 2 min 35×; 72°C for 10 min. The PCR product was 1755 nt for *Robbea* sp.2 and 1783 nt for *Robbea* sp.3.

### DNA extraction and PCR amplification of 16S rDNA

Symbionts were washed off a deep-frozen pellet of 500 *Robbea* sp.2 individuals with 50 µl of ddH_2_O. The 50 µl was then transferred to a fresh 1.5 ml tube and incubated at 94°C for 10 min. Five microlitres of this solution was directly used as a template for PCR. For *Robbea* sp.1 and *Robbea* sp.3, DNA was extracted from single worms as described (Schizas *et al.*, 1997), and 2 µl each was used as a template for PCR. For all *Robbea* worms, PCR was performed using the eubacterial primers 616V (5′-AGAGTTTGATYMTGGCTC-3′; Juretschko *et al.*, 1998) and 1492R (5′-GGYTACCTTGTTACGACTT-3′; Kane *et al.*, 1993). The PCR programme for *Robbea* sp.2 and sp.3 was: 94°C for 5 min; 94°C for 45 s, 47°C for 45 s, 72°C for 1 min 30 s 35×; 72°C for 10 min. Cycling conditions for *Robbea* sp.1 were: 94°C for 4 min; 94°C for 45 s, 49°C for 30 s, 72°C for 1 min 45 s 35×; 72°C for 10 min. Each PCR product was 1499 nt.

### DNA extraction and PCR amplification of APS reductase (*aprA*) gene

We extracted and purified the DNA from single *Robbea* worms as described previously (Schizas *et al.*, 1997) and 2 µl was used as a template for each PCR. To amplify a *c*. 1400 nt *aprA* (adenosine phosphosulfate reductase alpha subunit) gene fragment we used the primers AprA-1-FW (5′-TGGCAGATCATGATYMAYGG-3′) and AprA-10-RV for *Robbea* sp.1-associated bacteria (5′-CKGWAGTAGWARCCRGGRTA-3′) and AprA-11-RV (5′-CKGYRRTAGTAKCCSGGCCA-3′) for *Robbea* sp.2- and *Robbea* sp.3-associated bacteria, as described (Meyer and Kuever, 2007a,b).

### Cloning

All PCR products were gel purified and cloned into pCR2.1-TOPO using the TOPO TA Cloning Kit (Invitrogen Life Technologies, Germany).

We randomly picked and fully sequenced: 8, 7 and 6 clones of the 18S rDNA fragments obtained by *Robbea* sp.1 (EU768870), sp.2 (EU76887) and sp.3 (EU784735) respectively; 13, 19 and 11 clones of the 16S rDNA fragments obtained by *Robbea* sp.1 (EU711427), sp.2 (EU711426) and sp.3 (EU711428) respectively; 24, 31 and 21 clones of the *aprA* gene fragment from *Robbea* sp.1 (EU864035), sp.2 (EU864037) and sp.3 (EU864039), respectively. Sequences were aligned and compared with CodonCode Aligner 1.6.3 software.

### Phylogenetic analysis

For each *Robbea* species, the sequences of the symbiont 16S rDNA and the worm 18S rDNA were compared with sequences in GenBank by using blastn, the AprA sequences by using blastp (Altschul *et al.*, 1990). Phylogenetic analysis was carried out using the arb program package (Ludwig *et al.*, 2004). We used TreePuzzle 5.0 to evaluate the phylogenetic position of each *Robbea* worm and its respective symbiont. For 18S rDNA-based phylogenetic reconstruction, we also used the maximum parsimony method and constructed a consensus tree. Similarity matrices were calculated using the similarity matrix option in the neighbour joining field of the arb software package.

For tree calculations, we applied a 50% conservation filter and we used only sequences longer than 1450 bp for host phylogeny and longer than 1325 bp for symbiont phylogeny. Sequences of *Priapulus caudatus* (AF025927) and *Halycriptus spinulosus* (AF342790) for the host 18S rDNA tree and sequences of *Alkalimnicola halodurans* (AJ404972), *Nitrococcus mobilis* (L35510) and *Methylohalobius crimeensis* (AJ581837) served as out-groups for the symbiont 16S rDNA tree.

For the AprA protein tree, we aligned selected members of SOB, sulfate-reducing prokaryotes (SRP) and the stilbonematid symbiont sequences using T-coffee (Notredame *et al.*, 2000). We applied a 50% conservation insertion deletion (indel) filter for tree calculation and members of the AprA lineage I (Meyer and Kuever, 2007b) served as out-groups.

### Fluorescence *in situ* hybridization (FISH)

We designed FISH probes by using the ARB PROBE_DESIGN tool (see Table 1) and confirmed their specificity by comparing them with all available sequences in GenBank, SILVA, Greengenes. Probes were fluorescently labelled on their 5′ end (Thermo, Germany). FISH was performed according to Manz and colleagues (1992). Briefly, fixed nematodes (*n* = 30) of each *Robbea* sp. were incubated at 46°C in hybridization buffer containing the respective FISH probes [0.9 M NaCl, 20 mM Tris·HCl (pH 8.0), 0.001% SDS; refer to Table 1 for incubation time, formamide percentage and probe concentration]. Unspecific bound probe was subsequently removed by incubating at 48°C for 15 min in appropriate washing buffer. Nematodes were mounted in DAPI Vectashield (Vector Labs) and examined using a Leica TCS-NT confocal laser scanning microscope.
